# Effect of nutritional promotion intervention on dietary adherence among type II diabetes patients in North Shoa Zone Amhara Region: quasi-experimental study

**DOI:** 10.1186/s41043-023-00393-3

**Published:** 2023-05-30

**Authors:** Akine Eshete, Abera Lambebo, Sadat Mohammed, Sisay Shewasinad, Yibeltal Assefa

**Affiliations:** 1 Department of Public Health, Debre Berhan University, Debre Brehan, Ethiopia; 2 Department of Pediatric and Child Health Nursing, Debre Berhan University, Debre Brehan, Ethiopia; 3 School of Public Health, The University of Queensland, Brisbane, Australia

**Keywords:** Nutritional Health Promotion Program, Diet adherence, Patients with type II diabetes, North Shoa Zone Amhara Region

## Abstract

**Background:**

Diabetes is a major global public health problem that requires self-management behavior. However, this is difficult to implement in practice and requires new approaches. The purpose of this study was to evaluate nutritional promotion interventions for dietary adherence and lessons learned to improve self-management.

**Methods:**

A quasi-experimental study was conducted from January 2020 to February 2021 in North Shoa Zone public hospital. The study enrolled 216 type II diabetic patients from four public hospitals. Study participants were randomly assigned to intervention and control groups at an individual level. Data were measured twice (baseline and end line survey after six months using interviewer-administered questionnaires). Data were entered into Epi Data V.3.1 and analyzed using SPSS version 22. Data were presented as means of standard deviations for continuous variables and percentages for categorical variables. Intervention and control groups were compared before and after intervention using independent *t* tests. A *p*-value less than 0.05 was considered significant for all statistical tests.

**Results:**

A total of 216 type II diabetics participated in this study. Nutritional promotion intervention programs increased adherence to the mean number of days adhering to a healthy diet (*p* < 0.0001). Specifically, the nutrition promotion program improved daily intake of fruits and vegetables, low glycemic index foods, high fiber foods, healthy fish oils, low sugar foods, and healthy eating plans (*p* ≤ 0.050). Mean fasting blood glucose levels were significantly decreased after the educational intervention (*p* ≤ 0.05).

**Conclusion:**

This study demonstrates that a nutrition-promoting intervention can significantly change patients' adherence to healthy eating behaviors and effectively improve their glycemic control. Health care providers should integrate programs that promote nutrition education into existing health systems service. Primary care platforms such as health posts and health centers can play a key role in integrating health promotion programs to improve self-management behaviors.

## Background

Diabetes is a major public health problem worldwide [1], with a prevalence of 9.3% in 2019 and rising to 12.2% in 2045 [2–5]. Ethiopia has the highest prevalence of diabetes, ranging from 2.0% to 6.5% [6]. This rapid increase in diabetes requires self-management behavior, especially in areas with poor health care facilities. Diabetes self-management includes activities and behaviors that patients undertake to manage and treat their condition [7]. Diabetes requires ongoing care with multifactorial risk reduction strategies. According to the American Diabetes Association (ADA), successful diabetes management requires a systematic approach that supports patient behavior change efforts [8].

Nutrition education is effective in improving nutritional knowledge [9], but nutritional practice is the most common challenge in the management of type II diabetes [8, 10, 11]. To address this issue, people with diabetes should be actively engaged in nutritional interventions [8, 10, 11].

Successful nutritional interventions require behavioral changes that support adherence to nutritional interventions [12]. Successful nutrition education is strongly associated with selection and adherence to recommended dietary recommendations [13, 14] and has been shown to improve eating behavior and clinical outcomes [15, 16]. Previous studies have shown that nutrition education is significantly associated with adherence to recommended dietary habits in several countries [17–24]. On the other hand, poor nutritional knowledge and conflicting dietary recommendations can lead to poor dietary adherence [25].

Various studies in Ethiopia found adherence to dietary recommendations ranged from 25.7% to 53.2%. The findings indicate that many patients adhere to limiting intake and a healthy diet to prevent and treat type II diabetes [26–31].

Efforts to promote diabetes self-management behaviors are therefore a priority for Ethiopia. One of the common goals of medical nutrition therapy and diabetes self-management education is to improve diabetes-related nutritional knowledge to facilitate nutritional practice. Therefore, the aim of this study was to assess the effects of nutritional promotion interventions on dietary adherence and glycemic control in her type II diabetic patients in the study area. Furthermore, this study provides relevant information for making evidence-based decisions and designing appropriate community interventions, as well as planning and designing future behavioral promotion strategies and interventions. Additionally, the results of this study will help people with diabetes and their healthcare providers plan appropriate interventions to ensure optimal health.

## Methods

### Study area and period

The study was conducted in North Shoa Zone public hospitals from January 2020 to February 2021. North Shoa is one of the thirteenth zones of the Amhara Regional State located in northern Ethiopia. It has 24 districts and three city administrations. There are thirteenth hospitals and all public hospitals have diabetic follow-up services.

### Study design

A quasi-experimental study was conducted in a randomly selected public hospital. A nutritional intervention was implemented for six months after relevant baseline data were collected from the intervention and control groups. The study included interventions focused on recommended portion sizes, healthy eating plans, balanced meals and portions, and counseling about unhealthy food intake.

### Subjects and sample selection

A total of 216 eligible participants were enrolled in the study. Five hundred eight patients were excluded from the study for various reasons. These included 294 patients with other types of diabetes, 97 patients with less than three months of follow-up, and 117 patients with serious complications. The study included consenting patients, aged 20 to 70, with no complications, who stayed for at least six months, and had no intention of leaving. Patients who had other types of diabetes, patients who had disease duration of less than 6 months, refused consent, patient who were unable to participate in interventions based on physician assessment (e.g., acute illness, mental illness, and dementia) and patients with severe visual impairment were excluded from the study.

All samples (216) were divided into intervention (108) and control (108) groups assuming an equal sample distribution. Intervention and control groups of diabetic patients were selected from different locations within the zone. In all groups, participants were matched with age, body max index (BMI), and gender, and had fasting blood glucose levels > 126 mg/dL (7.0 mmol/L) [8, 32].

Study participants were registered under a specific code but were not informed of their group assignment and thus were unaware of differences between the intervention and control groups. Study participants were randomly assigned to intervention and control groups at an individual level. The list of participants and their codes are kept only by the researcher. All groups of study participants were geographically separated to avoid the risk of contamination. Additionally, patients, health care providers, and promoters (health educators) were blinded to the study results to avoid the hawthorn effect.

### Implementation and follow-up of intervention

After collecting relevant baseline data from both groups, a physical activity promotion program was implemented for a period of six months. Two health promoters and one facilitator were recruited for the intervention group and trained on the implementation and packaging of physical activity promotion of program modules. Training focuses primarily on session structure, communication skills and style. In addition, health promoters were trained in educational modules.

In addition, health promoters were trained in educational modules. Educational modules have been developed based on recommended international guidelines and local practices [5, 8, 32–34]. Module content was approved by an expert panel of physicians, nutritionists, and health educators.

Once the educational module was validated, the intervention was offered to participants over a period of 6 months. The first and second intervention sessions focused on the overview and basic concepts of DM, including risk factors, diagnosis, symptoms, course, diabetic complications, and treatment of type II patients. The goal of this session was to create a sense of urgency in the patient to take action.

The 3rd and 4th sessions will cover type 2 diabetes mellitus management based on national and international guidelines [8, 13, 32, 35, 36], including a varied and balanced diet, how to reduce unhealthy eating habits, and how to replace foods not recommended for people with diabetes with recommended foods, healthy eating habits, how to prepare and cook healthy food. The purpose of this session was to encourage behavior change. The fifth and sixth intervention sessions focused on adherence to recommended dietary recommendations, development of a healthy eating plan, and clinical monitoring of glycemic control. The goal of this session was to focus on lifestyle change counseling and encourage long-term commitment to behavior change. At the end of each session, health promoters and participants consolidated the components of the intervention session to develop a personalized action plan for maintaining diabetes care.

Patients attended an educational session on the same day as their medication appointment. Three educational sessions were held each week. Educational sessions take the form of lectures, group discussions and sometimes individual consultations. During follow-up, patients received written educational materials to help with practical management.

A control group did not receive any specific intervention during follow-up. Control patients received usual care according to national guidelines for non-communicable diseases. During follow-up, the study group was adhere to the patient's care by the healthcare provider and study team as in the intervention group, except for the newly developed exercise package. All patients at the hospital received similar exercise package after the newly introduced package was scale up.

### Measurement of the outcome variables

Outcome parameters included changes in dietary practice and changes in glycemic control levels. Data were measured twice (baseline and end line survey after six months using interviewer-administered questionnaires). At each selected public hospital, two trained nurses collected all relevant data. During follow-up, the patient's blood glucose was measured with a PRODIGY® blood glucose meter.

Dietary data were collected by asking participants nine health questions based on the Perceived Dietary Adherence Questionnaire (PDAQ) [37]. Responses were recorded in days based on a 7-Likert scale. Higher values indicate higher compliance. Patients who reported 4 to 7 days were classified as practicing good eating habits, while, less than four days were classified as poor dietary practices.

Fasting blood sugar (FBS) is the most commonly used measure of glycemic control. The patient was asked to fast at least 8 h prior to the appointment in order to provide a blood sample for laboratory testing. The mean of the FBS measurements for three consecutive months was used for the baseline FBS analysis. Mean values ​​of six consecutive months of FBS measurements were used for analysis during follow-up. Based on ADA guideline recommendations, glycemic status is classified as good if mean FBS is between 80 and 130 mg/dL and poor if above 130 mg/dL [32].

### Data management and statistical analysis

For statistical analysis, data were entered into Epi Data version 3.1 and exported to the Statistical Package for Social Sciences (SPSS) version 22. Continuous variables presented as the mean ± standard deviation and categorical variables presented as percentages. Intention-to-treat analysis was used to test the hypothesis. Comparisons between groups were performed using independent-samples *t* tests to assess the effects of interventions on dietary habits. *p*-values ​​less than 0.05 were considered significant for all statistical tests.

## Results

### Socio-demographic and clinical characteristics of the study participants

**Fig. 1:**
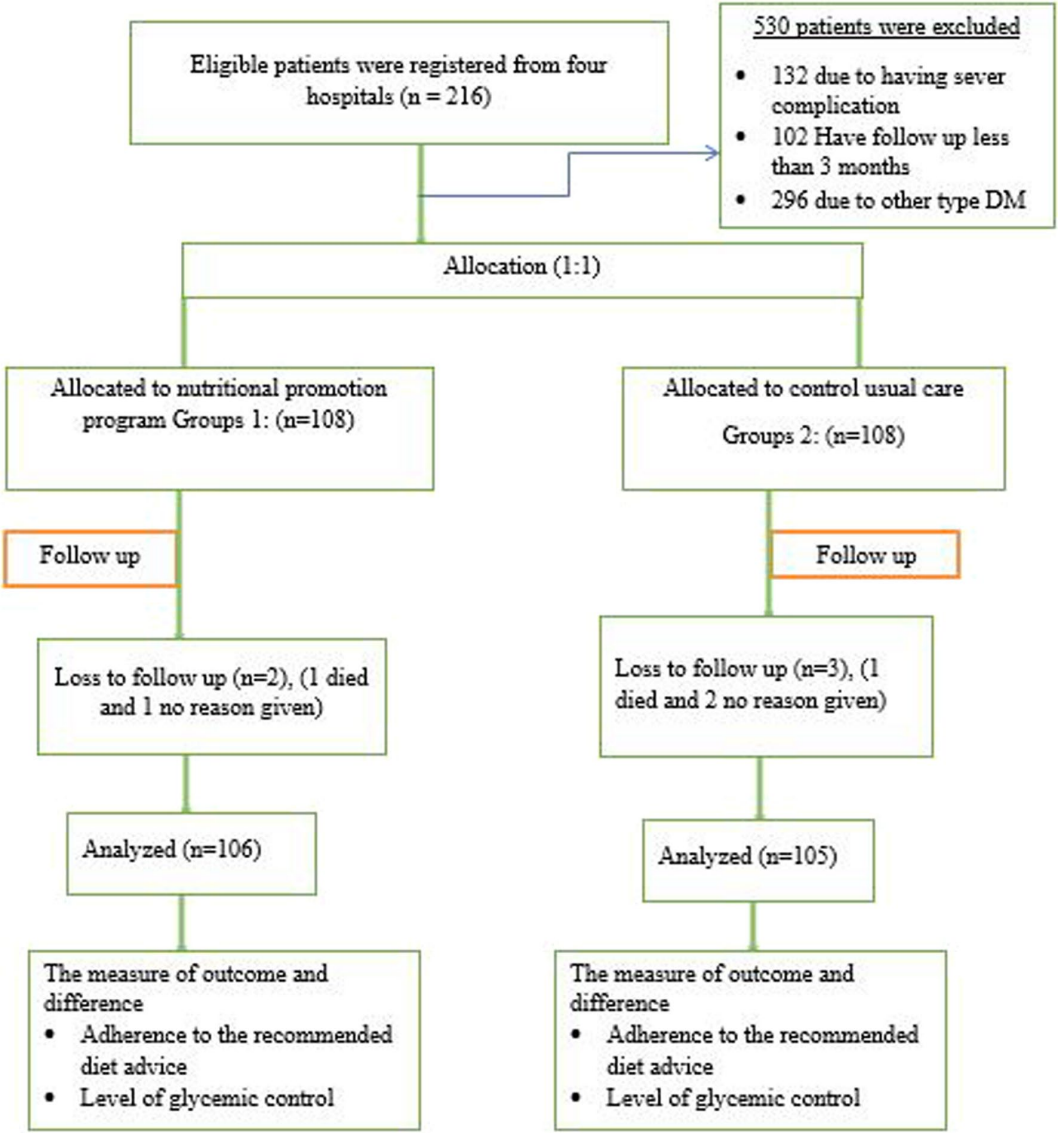
Flow of participants in the study

A total of 216 type II patients participated in the study. The majority of study participants in both groups were male, 60 (55.6%) and 56 (51.9%), being married, 71 (65.7%) and 99 (91.7%), and aged 48 to 63 years, respectively. Most of the study participants in both groups worked as farmers. Of the 216 patients, 57 (52.8%) in the intervention group and 55 (50.9%) in the control group were from rural areas. Moreover, 74 (68.5%) of the participants in the intervention group and 45 (41.7%) participants in the control group had no formal education (Fig. 1).

In both groups, 63 (58.3%) in the intervention group and 30 (27.8%) in the control group had comorbidities. The mean duration since diagnosis of DM was 5.14 ± 3.5 years and 4.19 ± 2.4 years in intervention and control groups, respectively (Table 1).

**Table 1:** Baseline socio-demographic and clinical characteristics of the patients in the North Shao zone, 2021

Questions	Intervention (*n* = 108, %)	Control (*n* = 108, %)
Age of the respondent		
Young age group (15–47 years)	31 (28.7)	34 (31.5)
Middle age group (48–63 years)	57 (52.8)	62 (57.1)
Elder age group (≥ 64)	20 (18.5)	12 (11.1)
Sex of the respondent		
Male	60 (55.6)	56 (51.9)
Female	48 (44.4)	52 (48.1)
The residential area of respondents		
Urban	51 (47.2)	53 (49.1)
Rural	57 (52.8)	55 (50.9)
Current marital status of the respondent		
Married	71 (65.7)	99 (91.7)
Other (Single, Divorced, Widowed)	37 (34.3)	9 (8.3)
Educational status of the respondent		
No formal education	74 (68.5)	45 (41.7)
Attended formal education	34 (31.5)	63 (58.3)
Employment status of the respondents		
Farmer	53 (49.1)	35 (32.4)
Housewife	28 (25.9)	34 (31.5)
Other (Government employee, Private employee, and Merchant	27 (25.0)	39 (36.1)
Duration since diagnosis of DM (years), (Mean ± SD)	5.14 ± 3.5	4.19 ± 2.4
Duration since starting DM treatment (years), (Mean ± SD)	4.7 ± 3.6	4.18 ± 2.4
Family history (Yes)	49 (45.4)	20 (18.5)

### Effect of a nutritional promotion intervention program on the intake of recommended diet items

The mean score intake of fruits and vegetables, low glycemic index foods, restricted or recommended foods, fish, healthy oils, and recommended oils in diabetic patients and high-fiber food groups increased after nutritional promotion intervention in the intervention group compared with the control group, and there was a statistically significant difference between groups (*p* < 0.05). In the mean scores of the high-fat food, nutrition education did not show a statistically significant difference between the two groups during the study period (*p* = 0.842) (Table 2).

**Table 2:** Comparison of the mean intake score of the recommended food items in the intervention and control group in North Shao zone, 2021

Recommended diet items	Type of group	After 6 months follow up	Independent *t* test			
			Mean difference	*t*	*df*	*p*-value
Followed a healthy fully eating plan	Intervention	4.23 ± 1.93	1.55 ± 0.23	6.8	184.6	< 0.0001
	Control	2.68 ± 1.30				
Fruits and vegetables	Intervention	2.80 ± 0.86	0.24 ± 0.13	1.9	209	0.004
	Control	2.56 ± 0.98				
Low GI	Intervention	3.26 ± 1.32	0.53 ± 0.18	3.0	204	0.003
	Control	2.73 ± 1.27				
High sugar foods	Intervention	0.87 ± 1.11	− 0.38 ± 0.16	− 2.3	209	0.020
	Control	1.25 ± 1.24				
Eat restricted or recommended food	Intervention	2.87 ± 1.23	0.56 ± 0.17	3.4	209	0.001
	Control	2.31 ± 1.22				
High fiber foods	Intervention	2.41 ± 1.16	0.93 ± 0.16	5.7	209	< 0.0001
	Control	1.48 ± 1.19				
High-fat foods	Intervention	1.12 ± 2.17	0.06 ± 0.28	0.2	209	0.842
	Control	1.07 ± 1.89				
Fish (servings/week)	Intervention	1.19 ± 0.72	0.66 ± 0.11	6.1	200.4	< 0.0001
	Control	0.52 ± 0.88				
Healthy oils	Intervention	2.75 ± 2.26	0.80 ± 0.32	2.5	209	0.012
	Control	1.94 ± 2.34				
The overall dietary mean score	Intervention	2.38 ± 0.65	0.55 ± 0.09	6.3	209	< 0.0001
	Control	1.83 ± 0.61				

### Adherence to the recommended food diet

**Fig. 2:**
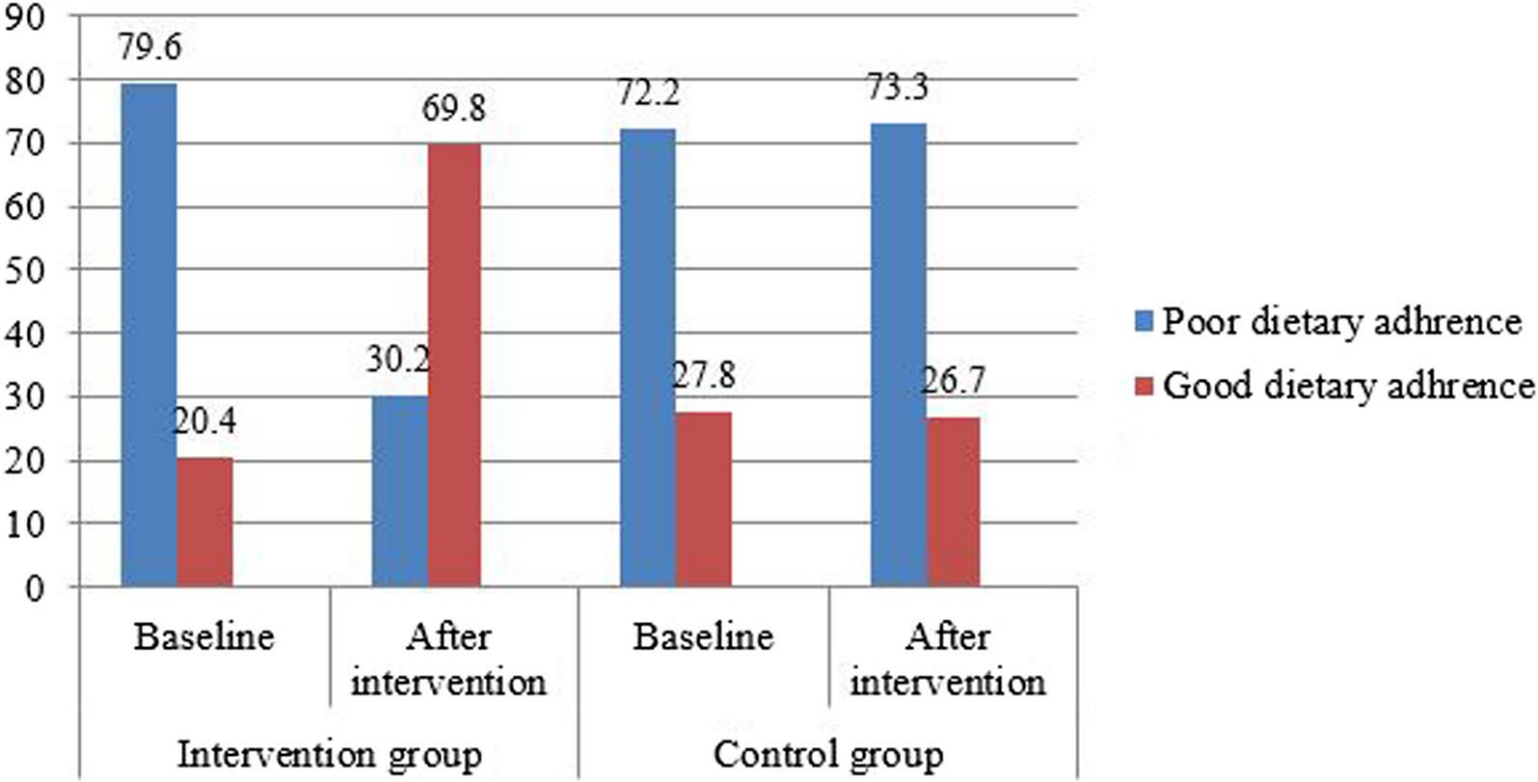
Proportion of dietary adherence to the recommended diet advice among the two groups in the North Shoa Zone, 2021

After 6 months follow-up, the proportion of good dietary adherence in the intervention group was 74 (69.8%), which was higher than the control group 28 (26.7%) (Fig. 2). The overall dietary mean score of the recommended diet was higher in the intervention group than in the control group, 2.38 ± 0.65 and 1.83 ± 0.61, respectively and with statistically significant differences between groups by independent *t* test after the educational intervention (*p* < 0.001) (Table 2).

### Effect of nutritional promotion interventional program on glycemic control

**Fig. 3:**
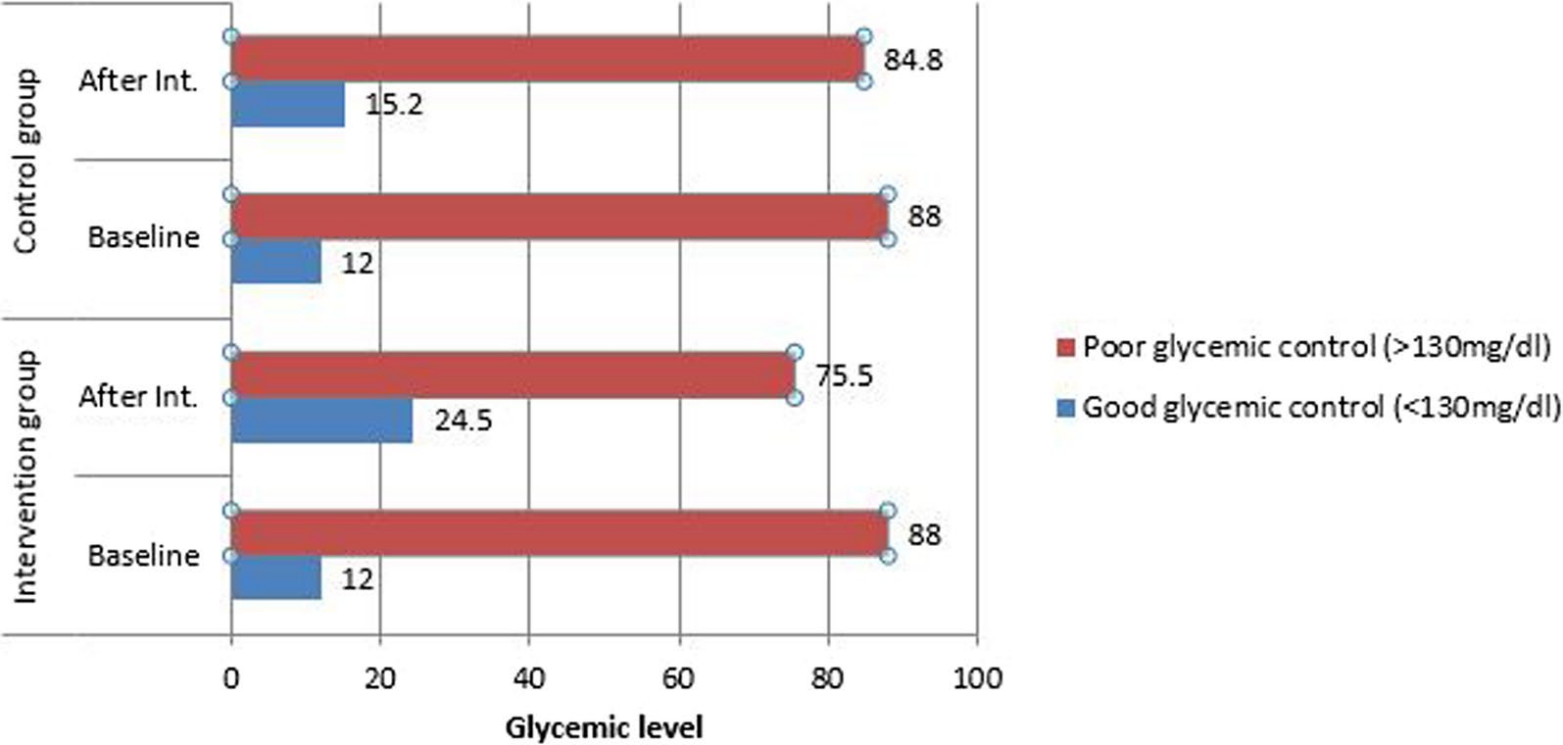
Proportion of glycemic control among the two groups in the North Shoa Zone, 2021

Mean fasting blood glucose after the nutritional promotion intervention was 172.14 ± 54.81 mg/dl in the intervention group and 186.64 ± 54.95 mg/dl in the control group. After the educational intervention, mean fasting blood glucose scores were statistically significant between groups in independent tests (*p* ≤ 0.05) (Table 3).The proportion of patients with good glycemic control was 26 (24.5%) in the intervention group and 16 (15.2%) in the control group after the 6-month intervention (Fig. 3).

**Table 3:** Comparison of the mean score of FBS in the intervention and control groups in the North Shoa zone, 2021

Variable	Type of group	Mean ± SD		A paired *t* test (*p*-value)
		Before the intervention	After 6 months follow up	
FBS (mg/dl)	Intervention	199.2 ± 76.57	172.13 ± 54.82	0.005
	Control	191.56 ± 52.79	186.38 ± 53.15	0.359
	Independent *t* test (*p*-value)	–	*p* < 0.050	–

## Discussion

This current study aimed to assess the effects of a nutritional promotion intervention program on adherence to recommended diets and glycemic control. In this study, the nutritional intervention program significantly increased the overall mean number of days adhering to a healthy diet. After the educational intervention, the mean days of adherence to a healthy diet increased in the intervention group compared to the control group, with a significant difference (*p* < 0.0001). This finding was supported by previous studies [13, 16, 38, 39], which indicate that nutrition education aims to improve dietary habits.

In this study, the mean of following a healthy diet plan in the intervention group increased after the educational intervention from 2.02 ± 0.66 to 4.23 ± 1.93. There was a statistically significant (*p* < 0.0001) difference between groups in independent sample testing. The results of this study demonstrate that educational interventions have a positive effect on adherence to a healthy eating plan. There is evidence that good meal plans can be helpful in resource-poor settings because they are simple, convenient, and promote comprehension and memory [13]..

In this current study, mean fruit and vegetable intake was higher in the intervention than in the control group, with statistical significance (*p* ≤ 0.050). The results of this study were similar to previous studies [39, 40], with significant improvement after intervention. Therefore, the results indicated that educational interventions were effective in increasing the number of days of fruit and vegetable consumption. Fruits and vegetables have been shown to play an important role in reducing the risk of various complications [35, 41], so increasing fruit and vegetable consumption in DM patients is recommended.

In this current study, mean day scores on low glycemic index foods was increased in the intervention group compared to the control group, showing a statistically significant difference (*p* = 0.003). Mean days on a high-fiber diet increased in the intervention group compared to the control group, showing a statistically significant difference (*p* < 0.0001). Consistent results were observed in studies conducted in Kenya [39] and Japan [42]. There has been evidence that a high-fiber diet may help manage diabetic complications. Soluble dietary fiber slows the absorption of glucose from the small intestine and prevents postprandial rises in blood glucose [35].

In this study, the average number of days consuming healthy oil and fish was increased in the intervention group compared to the control group. It was less than the recommended intake of fish per serving. Based on national and international guidelines, three to four servings of fish per week are recommended as part of a healthy, balanced diet [35].

This study demonstrates the effect of a nutrition promotion program on glycemic control. As this recent study shows, nutrition education has shown significant improvements in glycemic control. Based on the results, mean fasting blood glucose levels were significantly decreased after the educational intervention (*p* < 0.05). This finding is consistent with previous studies [16, 43–46], which indicated that FBS levels were significantly reduced after the educational intervention.

This study has the following limitations: Findings were based on patient responses to assessments of dietary changes that contribute to information bias. A social desirability bias may have influenced the results. Moreover, having multiple education methods means that you can't know which method made a difference in the study group. Additionally, we do not use propensity scores to control and minimize selection bias and confounders because different covariates were observed between groups. A propensity score method is recommended to minimize selection bias for estimating the impact of interventions on dietary changes. Propensity scores are used to balance program and comparison groups based on a set of baseline characteristics.

### Implication of the study

Implementation of nutrition promotion programs is the preferred strategy in diabetes management. This has been shown in previous studies [13, 16, 38, 39] and in this study. To support patient self-management behaviors, recommended diabetes interventions should be integrated into community-based health care and take into account the patient's cultural context. Therefore, primary care platforms such as health posts and health centers can play an important role in integrating health promotion programs to improve self-management behaviors. Comprehensive and timely patient-centered intervention strategies are needed to improve self-management behavior at the community and household level. This can be applied by integrating diabetes management interventions into community health services (health extension packages).

In summary, policy makers and providers are focusing on the following key program areas:

1) Nutritional health promotion programs should be integrated into existing systems as a common therapeutic service/treatment/. 2) Design different strategies to create health promotion programs to reach large communities in need using a wide range of learning strategies. 3) Assign health promoters to provide and design health education programs on nutritional advice recommended as routine care. 4) Focus on detailed analysis and understanding of the barrier faced by the patient and integrate them into their daily activities; additionally, providers should understand the basic nutritional needs of their patients.

## Conclusions

This study found that a nutritional intervention program could significantly increase the overall mean number of days of adherence to a healthy diet and improve glycemic control in patients. After the educational intervention, the mean days of adherence to a healthy diet increased in the intervention group (2.38 ± 0.65) compared to the control group (1.83 ± 0.61), with a significant difference (*p* < 0.0001). Specifically, mean days of fruit and vegetable consumption were higher in the intervention group (2.80 ± 0.86) than in the control group (2.56 ± 0.98), showing statistical significance (*p* < 0.004). The nutrition education program had significant improvement in the mean weekly consumption of low glycemic index foods (*p* < 0.001), high fiber foods (*p* < 0.001), fish (*p* < 0.001), healthy oils (*p* < 0.012), and improved in healthy eating plan (*p* < 0.001).

However, this intervention did not significantly improve high-fat diet consumption between groups (*p* = 0.842). Furthermore, mean fasting blood glucose levels were significantly lower after the educational intervention, with a significant difference (*p* < 0.050). Mean fasting glucose test level was 172.14 ± 54.81 mg/dl in the intervention group and 186.64 ± 54.95 mg/dl in the control group.

## Data Availability

All data generated in this study are included in the manuscript. Datasets are available upon reasonable request from the corresponding author.

## Abbreviations

ADA: American Diabetes Association
DM: Diabetes mellitus
FBS: Fasting blood glucose
GI: Glycemic Index
PDAQ: Perceived Dietary Adherence Questionnaire
SPSS: Statistical Package for Social Sciences
T2DM: Type II diabetes mellitus
